# Detection of Subclinical Motor Speech Deficits after Presumed Low-Grade Glioma Surgery

**DOI:** 10.3390/brainsci13121631

**Published:** 2023-11-24

**Authors:** Vesna Mirkoska, Malin Antonsson, Lena Hartelius, Fredrik Nylén

**Affiliations:** 1 Speech and Language Pathology Unit, Institute of Neuroscience and Physiology, Sahlgrenska Academy at the University of Gothenburg, 40530 Gothenburg, Sweden; malin.antonsson@neuro.gu.se (M.A.); lena.hartelius@neuro.gu.se (L.H.); 2 Department of Clinical Sciences, Umeå University, 90736 Umeå, Sweden

**Keywords:** low-grade glioma, motor speech, diadochokinesis, acoustic analysis

## Abstract

Motor speech performance was compared before and after surgical resection of presumed low-grade gliomas. This pre- and post-surgery study was conducted on 15 patients (mean age = 41) with low-grade glioma classified based on anatomic features. Repetitions of /pa/, /ta/, /ka/, and /pataka/ recorded before and 3 months after surgery were analyzed regarding rate and regularity. A significant reduction (6 to 5.6 syllables/s) pre- vs. post-surgery was found in the rate for /ka/, which is comparable to the approximate average decline over 10–15 years of natural aging reported previously. For all other syllable types, rates were within normal age-adjusted ranges in both preoperative and postoperative sessions. The decline in /ka/ rate might reflect a subtle reduction in motor speech production, but the effects were not severe. All but one patient continued to perform within normal ranges post-surgery; one performed two standard deviations below age-appropriate norms pre- and post-surgery in all syllable tasks. The patient experienced motor speech difficulties, which may be related to the tumor’s location in an area important for speech. Low-grade glioma may reduce maximum speech-motor performance in individual patients, but larger samples are needed to elucidate how often the effect occurs.

## 1. Introduction

Low-grade gliomas are tumors arising from glial cells of the central nervous system. The peak incidence is found among young adults aged between 30 and 40 years [1]. The most common presenting symptoms are generalized or focal epileptic seizures. While neurological examinations mostly yield normal findings, extensive evaluations with greater sensitivity than standard tests have revealed subtle disorders affecting various cognitive functions [2]. Treatment options include surgical resection, radiotherapy, and chemotherapy in various combinations or sequences [3].

A common practice in the management of low-grade glioma is to assess cognitive domains including attention, executive function, non-verbal memory, and various language abilities pre- and postoperatively; the tasks included may be tailored based on tumor location [4]. In studies investigating language ability before surgery, reduced lexical retrieval has been reported as the most common language deficit [5,6,7,8]. Impairments to lexical retrieval acquired pre- or postoperatively have been found to persist or worsen at 3-month and 12-month follow-ups [7,8,9,10].

In studies that focused on language performance, motor speech deficits were mainly reported as incidental findings from the language evaluation or clinical examination. A handful of studies have reported dysarthria or disturbances in motor speech performance in patients with a tumor located in the middle and superior frontal gyri and in the opercular region [11,12]. In the immediate postoperative period, various degrees of dysarthria were reported after resection of tumors in the inferior, middle, and superior frontal gyri, more specifically in the supplementary motor area and the premotor cortex, as well as the opercular area [11,12,13,14,15]. In most of these studies, the speech difficulties resolved within 3 months, but deficits persisting for as long as 18 months after surgery were observed in Peraud et al. [12]. Despite such reports of motor speech deficits occurring before and/or after tumor resection, speech remains largely under-researched in patients with low-grade glioma. One caveat in interpreting these incidental reports is that while motor speech difficulties may indeed have a low prevalence in this population, the postoperative effect on speech might not be as easily observed. In a recent study by Latini et al. [16], the extent of resection was negatively correlated with the number of intra-tumoral spots related to speech articulation and speech output. Simply put, surgeons may opt to leave a residual tumor mass to avoid disrupting viable motor speech networks inside the tumor. In such cases, the counterfactuals may be difficult to evaluate.

Oral diadochokinesis (DDK) is a maximum-performance non-speech task that tests the speed and regularity of reciprocal lip, tongue, and jaw movements [17] and is considered especially effective in detecting subtle motor speech deficits that are not easily elicited in regular speech [18]. Studies have shown that syllable repetition is sufficiently sensitive to detect subclinical speech impairments in the prodromal stages of Huntington’s disease and in patients with mild traumatic brain injury [19,20]. DDK has been shown to have high diagnostic accuracy in differentiating between healthy controls and patients with amyotrophic lateral sclerosis, traumatic brain injury, primary progressive apraxia of speech, and Parkinson’s [18,20,21,22]. Although widely used in motor speech assessments, DDK is still a subject of debate. Some studies propose that there is insufficient empirical evidence that would support the clinical use of nonspeech parameters in analyzing motor speech in patients with neurological disorders [23], while others suggest that DDK performance mirrors speech proficiency [24].

The rationale of this paper is twofold. First, motor speech in low-grade tumors is a topic that has received little attention, and we wanted to address this under-researched issue. Second, we assumed that by stress testing the oral motor system, we might detect subclinical impairments that could go unnoticed in speech tasks. We explored motor speech performance in 15 patients with presumed low-grade glioma before and after resection by investigating the following research questions: (1) Does surgery for presumed low-grade glioma affect speech-motor proficiency? (2) How many patients have an impaired ability to produce rapid syllable repetitions relative to published norms due to the presumed low-grade glioma?

## 2. Materials and Methods

### 2.1. Participants

This study included 15 patients (mean age = 41.6 ± 14.6 years) diagnosed with presumed low-grade glioma for whom pre- and postoperative syllable-repetition recordings were available. Patients were recruited between November 2014 and September 2016 from the Department of Neurosurgery. The preliminary diagnosis made before the surgical intervention was based on magnetic resonance imaging scans, physical examination, and patient history. Tumor localization, contrast enhancement, and tumor volume were determined by a neurosurgeon using T2-weighted/FLAIR images and contrast enhancement from T1 with gadolinium. Language and speech mapping were performed preoperatively with nTMS and fMRI in patients planned for awake surgery. Three patients underwent awake surgery with consequent intraoperative language mapping. Gross total resection was performed on five patients, while seven patients underwent subtotal resection. The remaining three patients underwent partial resection. After surgery, tumor grading based on histopathological examination revealed that two patients had astrocytoma grade IV (glioblastoma), and two were diagnosed with oligondendroglioma grade III and oligoastrocytoma grade III. Since the inclusion criterion was presumed low-grade glioma, all 15 patients were included in the analysis. However, an additional analysis was made, excluding the patients with gliomas of grades III and IV. Exclusion criteria were moderate or severe developmental language or cognitive disorder, mother tongue other than Swedish, previous brain surgery, and/or other tumor treatments. Table 1 lists demographic and tumor-specific parameters for all participants enrolled in the study.

### 2.2. Data Collection

The data were collected during two test sessions, one prior to surgery and one 3 months afterward, when all participants underwent an extensive language assessment. The sessions lasted for between 2 and 3 h and were video recorded. Since the rapid syllable-repetition task was added to the test battery several months after the start of the (broader) project, it was administered to only 15 patients in a cohort of 32. The patients were instructed to repeat the syllables /pa/, /ta/, and /ka/ and the sequence /pataka/ as fast and evenly as possible on a single breath. On average, they performed the task twice per session, but they were given the opportunity to practice before performing it at the maximum rate. All participants were compliant and performed the test without difficulty. Information about tumor characteristics was obtained from patient records.

### 2.3. Data Processing

The syllable repetitions were extracted from the video recordings using MATLAB [25]. The start and end of syllable repetitions in the video recordings were identified manually. Of the 120 syllabic sequences expected—15 /pa/ + 15 /ta/ + 15 /ka/ + 15 /pataka/ before resection and 15 /pa/ + 15 /ta/ + 15 /ka/ + 15 /pataka/ after resection—3 (2.5%) were missing: /pa/ from patient ID16 and /ta/ from patient ID18 before surgery as well as /pa/ from patient ID18 after surgery. Hence, a total of 117 diadochokinetic (DDK) sequences were analyzed. The mean duration of the DDK sequences was 8.03 s (min = 1.50 s, max = 22.00 s).

### 2.4. Acoustic Analyses

The acoustic envelope has received recent attention as a promising domain for analyzing syllable sequences [26], and various automated algorithms have been developed to detect and study specifically DDK sequences using the envelope-based approach. Procedures in the Dysarthria Analyzer Project [27] may be applied to measure rate and regularity from an acoustic speech recording. Syllables are detected via unsupervised learning of spectral envelopes (Mel-frequency cepstral coefficients) while removing audible inspirations, non-speech sounds, and underperformed syllables using automated analysis of outliers. The accuracy of segmentation has been shown to be high when compared to manual segmentations in a very large dataset of 698 recordings [28]. We applied the method developed by Hlavnička [28] to analyze the rate and regularity of syllable repetitions, which has been indicated to have high reliability [28]. Median averaging of consecutive differences was employed to guard against the impact of any spurious misidentifications of syllables in the automatic procedure.

### 2.5. Acoustic Features

The acoustic features of interest were rate and irregularity. The number of syllables produced per second (the rate) was computed as the inverse of the median duration between consecutive voice onsets [28]. Irregularity was estimated as the median absolute deviation of durations as measured between consecutive voice onsets and expressed in milliseconds.

### 2.6. Statistical Analyses

The data were analyzed using the generalized estimating equations (GEE) method, which is suitable for comparing repeated measurements pertaining to the same participants [29]. In addition, GEE requires no imputation of missing data [30], which is useful considering the 2.5% missingness in the dataset. Separate analyses were performed for each of the four rapid syllable-repetition tasks. The significance level was set at *p* ≤ 0.05.

Participants’ syllable-rate scores were compared on both an individual and group level with normal data as published in Karlsson and Hartelius [31] and the Swedish Dysarthria Manual [32]. In Karlsson and Hartelius [31], the syllable rate was computed cumulatively across /pa/, /ta/, and /ka/ for male and female speakers, respectively, broken down into three age groups: <40 years, 41–60 years, and >60 years.

In that study, across the three syllables, females younger than 40 produced on average 6.8 (SD = 0.7) syllables/second, those between 41 and 60 years produced 6.1 (SD = 0.8) syllables/second, and those above 60 produced 5.8 (SD = 0.9) syllables/second, whereas males in the corresponding age groups produced 6.7 (SD = 0.9), 6.3 (SD = 0.9), and 6.1 (SD = 0.7) syllables/second, respectively. In the Swedish Dysarthria Manual [32], a single reference value for /pataka/ is provided. The reference value used for /pataka/ was 5.8 (SD = 1.0) syllables/second. Individual participants’ scores were compared with the values for the relevant sex and age group.

For the purposes of group level comparison in our study, mean rates and standard deviations across males and females and the age groups of <40 and 41–60 years published in Karlsson and Hartelius [31] were combined using the algorithm described in Higgins et al. [33]. Since only two patients (ID16 and ID34) were above 60, the age group of >60 years was not used in the computation; it would have unnecessarily increased the standard deviation and decreased the mean rate. An age-weighted combined rate of 6.4 syllables/second (SD = 0.9) was used as a reference across the monosyllable tasks, while the reference used for /pataka/ was the same as in the individual comparisons, 5.8 (SD = 1.0) syllables/second.

In the individual and group comparisons, 2 standard deviations (SD) below the mean were used as the cut-off for possible motor speech difficulties.

## 3. Results

**Figure 1:**
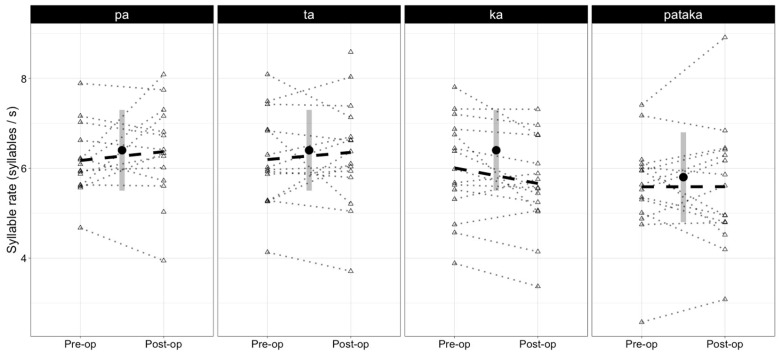
The median syllable rate achieved by each participant before (pre-op) and after resection (post-op). The dashed line indicates the group averages achieved in pre-op and post-op sessions. The single point with vertical segments at the center of each plot indicates the mean syllable rate, with standard deviations, reported in the literature for healthy speakers.

**Figure 2:**
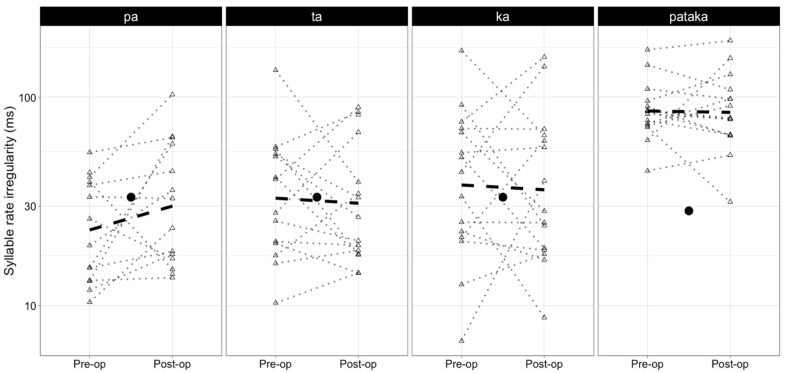
The median instability in syllable sequences produced before (pre-op) and after resection (post-op). The dashed line indicates the group averages achieved in pre-op and post-op sessions. The single point at the center of each plot indicates the average syllable rate and standard deviation reported in the literature for healthy speakers.

Figure 1 and Figure 2 illustrate the average rate and regularity of syllable repetition before resection and at 3-month follow-up. Table 2 shows the mean rate and regularity (with standard deviations, minimum, maximum, and median values) observed before and after resection for each syllabic sequence. In brief, the results of the present study showed a significant reduction in the rate of /ka/ production in pre-surgery (6.0 syllables/second) to post-surgery (5.6 syllables/second) evaluations but no significant effects on the regularity of syllable productions. No significant effects were observed for the /pa/ /pataka/, and /ta/ syllable sequences in terms of rate and regularity. Table 3 provides an overview of the results from the GEE model, representing the difference in rate and irregularity between the pre- and postoperative states.

On a group level, the participants’ syllable production rate was within the normal range relative to Swedish normative data on both assessment occasions across all syllable tasks. Although the rates for /ka/ and /pataka/ were slightly below average in healthy speakers, they were within the normal range both before and after surgery.

On an individual level, only one patient (ID 25) performed below the chosen cut-off across all syllable tasks on both assessment occasions. This patient’s median rate was 4.7, 4.0, 3.9, and 2.6 syllables per second for /pa/, /ta/, /ka/, and /pataka/ syllables, respectively, in the pre-surgical evaluation. In the post-surgical evaluation, the median syllable rates had decreased to 3.9, 3.7, and 3.4 syllables per second for /pa/, /ta/, and /ka/ syllables. The syllable rate of /pataka/ had increased to 3.1 post-surgery.

The results of the additional analyses, excluding the participants with glioma grades III and IV, mirror our previous findings. In short, a significant reduction in the rate of /ka/ production from pre-surgery (6.2 syllables/second) to post-surgery (5.9 syllables/second) was observed (95% CI [6.0, 6.4], *p* value = 0.02), but no significant effects on the regularity of syllable productions. No significant effects were observed for /pa/, /ta/, and /pataka/ syllable sequences in both rate and regularity.

## 4. Discussion

In our study, we addressed the possible effects of tumor resection on motor speech based on rapid syllable-repetition rate and regularity in patients with presumed low-grade glioma. Surgical resection seemed to be associated with a small reduction in the production rate for the /ka/ syllable but did not have any other group-level effects on the patients.

The postoperative decline observed in the rate for /ka/ might partly be explained by the articulatory demands of this syllable, which involve moving the dorsal part of the tongue to and from the velum. Although the muscles of the tongue are synchronized during speech, some studies have observed a higher level of activation in the genioglossus muscle during the articulation of the velar consonant [k] [34,35]. Unlike the other CN XII-innervated muscles of the tongue that have bilateral supranuclear innervation, the genioglossus receives primarily crossed and unilateral innervation, and this might make it more susceptible to damage [36,37]. Hence, the decrease by almost half a syllable per second may reflect a subtle change in motor speech performance. No control groups are reported here, but some normative data on the DDK rate is available in the literature. The decrease from 6 to 5.6 syllables/s is comparable to the reduced rate Karlsson et al. [31] observed in female speakers of 75 years of age compared to speakers that were 60 years old, or in male speakers of 82 years compared to 65-year-old speakers. Thus, the post-operative reduction in DDK rate observed here represents a subclinical decline in motor performance. In our study, we also observed a slower rate for /pataka/ before and after surgery. Some studies suggest that adults achieve higher rates of /pataka/ compared to same-syllable repetitions [24]. In that respect, when compared to the same syllable repetitions and against the reference value, the slower rate observed in this study might represent a subclinical disturbance of motor speech. The relatively low values observed for /ka/ and /pataka/ suggest that the repetition tasks involving them might be more sensitive to motor speech performance than those involving /pa/ and /ta/. Indeed, in Karlsson and Hartelius [22], regression models designed to differentiate between healthy speakers and Parkinson’s patients on the basis of syllable repetition showed that /ka/ had both better predictive accuracy (AUC = 0.93) and better sensitivity and specificity of prediction than either /pa/ or /ta/. There are no previous reference values for DDK irregularities that we could compare our findings to, so we discuss only the findings on syllable rate.

On an individual level, one patient with a fronto-temporal glioma (ID 25) performed below age-specific norms across all tasks and assessment periods. The patient’s preoperative deviation in syllable rate worsened somewhat postoperatively across the monosyllable sequences while remaining below the normal range for /pataka/, despite a small postoperative improvement. These findings imply that the patient had preoperative motor speech difficulties that persisted postoperatively. As regards the reasons for these difficulties, it should be noted that there are previous reports of speech deficits in the presence of frontal and opercular tumors, as well as reports of persistent speech difficulties following resection of glioma in those areas [11,12,13,14,15]. Although the impact of tumor location was not examined in our study, it appears that the reduced syllable rate might be related to the fronto-temporal location of the tumor. Specifically, the reason might involve a slowing down of the muscular adjustments required for syllable production. However, it might be somewhat misleading to attribute the slow rates solely to changes in speed because speed interacts with other neuromuscular features such as strength, tone, steadiness, range, and accuracy. Reduced or variable speed may be associated with variable range, tone, and accuracy of movement, suggesting that these features are rarely affected in isolation [17].

Finally, it is worth mentioning that, although statistically insignificant, some improvement was observed in the syllable rates of /pa/ and /ta/ on an individual and group level. At the same time, the rate of irregularity in the production of /pa/ increased. We believe that future studies with larger cohorts and a robust study design might focus on interpreting such relationships, if replicated.

## 5. Limitations and Future Directions

This study has several limitations. The first is the small and heterogeneous cohort, and in addition, 2.5% of DDK sequences were missing in the recordings. In small, underpowered studies, even if a true effect is detected, the estimate of its magnitude may be inflated. We used the GEE statistical method to alleviate the severity of the effect of missing data and allow evaluation of effects in all data that was available [29,38]. This is important in small-sample studies, as omitting subjects would further lower the reliability of the results. Finally, syllable repetition is only a part of the motor speech screening battery, and future studies should aim to administer the entire set of tasks. In our study, however, data were collected during an extensive and time-consuming language screening where syllable repetition was selected as a sensitive motor speech stress test that could reveal subclinical impairments that might go unnoticed in speech tasks.

Future studies should include a control group, a larger cohort of patients, and multiple measurements. It might be particularly interesting to follow patients with tumor residuals involving speech networks, as it might allow us to study any long-term effects. In that respect, the clinical implications should be established only after more extensive research. We believe that this topic deserves to be readdressed by the research community using more sophisticated study designs.

## 6. Conclusions

In this small sample of 15 participants, surgical resection of presumed low-grade glioma did not affect motor speech performance, as measured by syllable-repetition rate and regularity, except for the rate for the syllable /ka/, where a statistically significant postoperative decline was observed. Performance below normative data, indicating motor speech impairment, was found only for one participant. Despite the low prevalence of motor speech impairment found in this study, we suggest that screening of motor speech performance in patients with low-grade glioma using syllable repetition is a quick and non-invasive method that could add valuable insight into subtle speech deficits that may go undetected in standard language assessments. Syllable repetition could possibly also be a useful task to be administered during awake surgery, when it could provide real-time feedback on motor speech performance.

## Figures and Tables

**Table 1:** Demographic and clinical characteristics of patients.

ID	Sex/Age/ Education	Hd	Tumor Location	Lt	Volume (mL)/CE	Histology	Seizures	Previous Treatment	EOR	Post-op Treatment
12	M/31/22	R	Frontal/motor	L	45.1/N	A IV, GBM	Yes	No	2	RT, TMZ
16	M/62/14	R	Frontal	L	28.2/Y	OA III	Yes	R, chemo	3 A	SRS
23	M/55/16	R	Temporal/insula	L	10.2/F	A IV, GBM	Yes	R, RxT	2 A ^a^	TMZ
24	M/31/16	L	Insula	L	39.7/N	A II	Yes	R	3 PA	Proton
25	M/25/12	R	Fronto-temporal	L	27.2/D	O III	Yes	No	1	Proton. TMZ
32	M/53/13	R	Frontal/motor	L	86.8/N	O II	Yes	No	2	TMZ
36	M/24/12	L	Frontal	L	7.8/N	A II	No	No	2 A	No
20	F/42/12	R	Frontal/motor	R	1.4/N	A II	Yes	No	1	No
26	M/44/17	R	Frontal, temporal, insula, and thalamus/motor	R	150.3/F	O II	Yes	No	3	TMZ
18	F/49/12	R	Parietal	L	2.8/N	Ganglio-glioma I	No	No	1–2	No
19	M/26/19	R	Temporal	L	12.1/N	A II	Yes	No	1	No
27	M/56/15	R	Temporal	L	50.8/N	A II	Yes	No	2	No
29	M/26/16	R	Temporal	L	4.2/N	Ganglio-glioma II	Yes	No	2	No
34	M/67/17	R	Frontal	L	8.7/D	O II	No	No	1	No
17	F/34/15	R	Parietal	R	3.6/F	O II	Yes	R, R × T, chemo	1	No

Note. Sex: M = male; F = female. Education = number of years. Hd = handedness: R = right; L = left. Lt = lateralization of the tumor: R = right; L = left. CE = contrast enhancement; Y = yes; N = no; D = discrete; F = focal. Histology: OA = oligoastrocytoma; A = astrocytoma; O = oligodendroglioma; GBM = glioblastoma. Previous/postoperative treatment: R = resection; RT = radiotherapy; SRS = stereotactic radiosurgery; chemo = chemotherapy; TMZ = temozolomide; PCV = procarbazine, CCNU, and vincristine; Proton = proton-beam radiotherapy. EOR = extent of resection. (1) Gross total resection = 100%; (2) Subtotal resection = 90–100%; (3) Partial resection < 90%. A = awake surgery; PA = planned awake surgery. ^a^ The resection stopped because the patient found it hard to stay awake.

**Table 2:** Syllable rate and irregularity in patients with low-grade glioma before and after surgery.

		Rate		Irregularity	
		Pre-op	Post-op	Pre-op	Post-op
	min.	4.7	3.9	10.4	13.6
/pa/	mean (SD)	6.2 (0.8)	6.4 (1.1)	26.7 (14.6)	37.4 (26.8)
	median	6.0	6.4	22.8	28.1
	max.	7.9	8.1	54.4	103.0
	min.	4.1	3.7	10.3	14.3
/ta/	mean (SD)	6.2 (1.1)	6.4 (1.1)	41.0 (31.7)	38.7 (27.9)
	median	6.0	6.4	34.1	26.6
	max.	8.1	8.6	135.0	89.6
	min.	3.9	3.4	6.8	8.8
/ka/	mean (SD)	6.0 (1.1)	5.6 (1.1) *	51.0 (41.0)	50.0 (44.7)
	median	6.0	5.6	43.8	28.4
	max.	7.8	7.3	167.2	156.0
	min.	2.6	3.1	44.3	31.5
/pataka/	mean (SD)	5.6 (1.1)	5.6 (1.4)	90.2 (31.0)	92.3 (40.0)
	median	5.6	5.6	85.7	79.6
	max.	7.4	8.9	169.0	187.0

Note. Rate is expressed as the number of syllables per second and computed as the inverse of the syllable-duration median. Irregularity is expressed in milliseconds and computed as the median absolute deviation of durations as measured between consecutive voice onsets across the syllable sequence. Median rate and irregularity are computed on an individual level and then averaged across the cohort. Pre-op = before surgery; post-op = 3 months after surgery. N_(total)_ = 15; 2.5% missingness in the dataset. * = statistically significant difference pre- versus post-surgery. A weighted average of relevant age-specific norms was computed on the basis of data from Karlsson and Hartelius [31], and the result (6.4 syllables/second, SD = 0.9) was used as a reference for the participants’ performance of the /pa/, /ta/, and /ka/ syllable-repetition tasks. The reference value used for the /pataka/ task (5.8 syllables/second, SD = 1.0) was retrieved from the Swedish Dysarthria Manual [32].

**Table 3:** Results from the GEE analyses representing the difference in rate and regularity pre- and postoperatively.

		Rate				Irregularity		
	**/pa/**	**/ta/**	**/ka/**	**/pataka/**	**/pa/**	**/ta/**	**/ka/**	**/pataka/**
Intercept	6.11	6.31	6.005	5.59	26.08	41.66	51.03	90.23
Diff.	0.25	0.04	−0.346 *	0.003	11.77	−2.97	−1.09	2.04
Lower 95% of the diff.	5.68	5.95	5.73	5.19	13.48	23.17	26.04	74.28
Upper 95% of the diff.	6.54	6.68	6.28	5.98	38.69	60.15	76.03	106.18
*p* value	0.25	0.84	0.01 *	0.99	0.06	0.75	0.93	0.80

* *p* < 0.05; N_(total)_ = 15; 2.5% missingness in the dataset; Diff. is the difference in rate and regularity pre- and post-operatively. Rate is expressed as the number of syllables per second and computed as the inverse of the syllable-duration median. Irregularity is expressed in milliseconds and computed as the median absolute deviation of durations as measured between consecutive voice onsets across the syllable sequence. Median rate and irregularity are computed on an individual level and then averaged across the cohort.

## Data Availability

Data are unavailable due to our ethical restrictions.
